# Talk to the Virtual Hands: Self-Animated Avatars Improve Communication in Head-Mounted Display Virtual Environments

**DOI:** 10.1371/journal.pone.0025759

**Published:** 2011-10-12

**Authors:** Trevor J. Dodds, Betty J. Mohler, Heinrich H. Bülthoff

**Affiliations:** 1 Human Perception, Cognition and Action, Max Planck Institute for Biological Cybernetics, Tübingen, Germany; 2 Department of Brain and Cognitive Engineering, Korea University, Seoul, Korea; Royal Holloway, University of London, United Kingdom

## Abstract

**Background:**

When we talk to one another face-to-face, body gestures accompany our speech. Motion tracking technology enables us to include body gestures in avatar-mediated communication, by mapping one's movements onto one's own 3D avatar in real time, so the avatar is self-animated. We conducted two experiments to investigate (a) whether head-mounted display virtual reality is useful for researching the influence of body gestures in communication; and (b) whether body gestures are used to help in communicating the meaning of a word. Participants worked in pairs and played a communication game, where one person had to describe the meanings of words to the other.

**Principal Findings:**

In experiment 1, participants used significantly more hand gestures and successfully described significantly more words when nonverbal communication was available to both participants (i.e. both describing and guessing avatars were self-animated, compared with both avatars in a static neutral pose). Participants ‘passed’ (gave up describing) significantly more words when they were talking to a static avatar (no nonverbal feedback available). In experiment 2, participants' performance was significantly worse when they were talking to an avatar with a prerecorded listening animation, compared with an avatar animated by their partners' real movements. In both experiments participants used significantly more hand gestures when they played the game in the real world.

**Conclusions:**

Taken together, the studies show how (a) virtual reality can be used to systematically study the influence of body gestures; (b) it is important that nonverbal communication is bidirectional (real nonverbal feedback in addition to nonverbal communication from the describing participant); and (c) there are differences in the amount of body gestures that participants use with and without the head-mounted display, and we discuss possible explanations for this and ideas for future investigation.

## Introduction

Virtual environment (VE) technology allows multiple people to interact and communicate in a shared three-dimensional space. This paper addresses the communication aspect, which is important from both an applied and theoretical perspective. From the applied perspective, people want to communicate and are increasingly choosing VE technology to do so [1], [2]. Some people are choosing to use VEs to communicate instead of other technology simply because they are already in a VE: they require aspects of the VE (shared data, shared space) and need to communicate to collaborate on a given task. This can be seen in massively multiplayer online games, urban planning [3], and social systems such as Second Life [4]. On the other hand, VEs are a subset of communication media in general and can also be used for the sole purpose of telecommunication, e.g. [5].

From the theoretical perspective, virtual reality (VR) is a powerful medium for researching which elements contribute to naturalistic communication [6], [7]. Recent advances in technology enable us to do full-body motion tracking in real time, and map the movements onto self-avatars, e.g. [8], [9]. This is particularly useful for the study of nonverbal communication.

Nonverbal communication refers to aspects of communication that are not part of the words themselves, including facial expressions, body posture, and gestures [10]. It can also include people's outward appearance, for example, their height or the way they dress. A politician or business person would rarely be seen at an important meeting wearing jeans and a t-shirt: the suit they wear communicates something to those with whom they interact.

In VR we can control nonverbal communication and systematically manipulate it. We can ensure each participant wears exactly the same clothes by giving them the same avatar. We can make them the same height, and even give them the same face. We can then change one component to see its effect on communication. For example, Yee and Bailenson changed the height of participants' avatars and found that people with taller avatars negotiated more aggressively [11].

We identify three research questions for this work from the perspective of communication. First, how does the perspective of our avatar (e.g. first- vs. third-person) in head-mounted display (HMD) virtual reality affect communication? Second, is it important that our avatar is self-animated? Third, is it important that the ‘other’ avatar, the listener, is self-animated? Answering these questions will enable us to understand the effect of nonverbal feedback that listeners provide to their speakers, such as nodding the head when they are understanding, and changing gaze direction to indicate attention [12]. The effect of nonverbal feedback has implications for the development of computer-controlled virtual characters that attempt to implement feedback programmatically (e.g. using the techniques described in [13]). Finally, our work demonstrates how full-body motion tracking in virtual reality can be used for researching nonverbal communication by measuring of the rate of communication and the usage of gestures using a state-of-the-art motion tracking facility.

### Nonverbal communication

Nonverbal communication as defined above is broad in scope, (i.e. it can include facial expressions, eye gaze, appearance) and in this set of experiments we focus on the usage of gestures. Kendon's continuum identifies different types of gesture, from ‘gesticulations’ (body motions that naturally occur with speech) to conventionalized sign language [14]. We focus on the former type, and use the term ‘gestures’ to describe the movements of the hands that naturally occur with speech. This distinction is important because of the relation between gestures and speech. Linguistical properties of these gestures are low or absent entirely when accompanied by speech, and in contrast are present in sign language. The gestures we are investigating, therefore, should not be considered in the context of language, but as co-expressive with speech. And if McNeill is correct, the gestures are ‘co-expressive, but non-redundant’ [14].

We know that various types of nonverbal behavior are able to communicate something in addition to accompanying spoken words. Mehrabian showed that nonverbal communication influences the interpretation of positive, negative and neutral words, to the extent that only 7% of the interpretation was based on the words themselves. An example of this from everyday conversation is sarcasm, where the words may be positive but the alternative meaning is made clear from the way they are expressed nonverbally, i.e. with tone of voice and facial expressions [10]. Choi et al. give a review of nonverbal ‘leakage’, which refers to unintended communication that is expressed nonverbally. They argue that the encoding and decoding processes are largely automatic, to the extent that deceivers over-compensate in attempting to control their nonverbal leaks, therefore ironically arousing more suspicion [15]. Steptoe and colleagues show that these nonverbal cues can be successfully replicated using tracking technology with avatar-mediated communication, and the addition of nonverbals to VR increased participants' ability to detect lies [1].

Gestures also benefit the person speaking. For example, when people memorize a phrase using actions, they show an improvement in retrieving this information when they are physically performing the action [16]. Morsella and Krauss show that people gesture more when recalling objects that are ‘non-codable’ (abstract, without function), and when they are not visible at the time of recall [17]. This could suggest that in our experiments, speaking participants could be using gestures to help themselves recall information.

Determining to what extent gestures help communication by providing extra information to the listener, versus helping only the speaker, is not trivial, and arguing entirely for one side or the other is likely an oversimplification [14], [18]. For example, Rowe and Goldin-Meadow found that children's gestures can be used to explain an increase in their vocabulary, a result that on its own could be evidence that gestures are not communicating information, but helping the speaker with recall tasks. However, the use of gestures by the children was explained by the number of gesture types used by their parents as they communicated with their child. Ultimately, the gestures of the parents could explain the increasing vocabulary, and therefore gestures were both transmitting extra information from speaker to listener and aiding in recall [19]. In our set of experiments, it should be noted that whatever benefit speakers get from gesturing is available in all our experimental conditions. For although we manipulate participants' avatar representations (e.g. in one condition we make it static instead of self-animated), participants are still free to gesture as they please. Making the avatar static cuts off any communication benefit of the gestures for the person listening to them. It is of course possible, however, that manipulating the avatar (e.g. making it static) could cause participants to move less, which would then reduce the beneficial aspect for the speaker also. We return to this in the discussion of experiment 1.

### Responding to VR as if it were the ‘real world’

Our predictions are that (a) participants will manipulate their own avatars in the environment in a similar way to the real world, (i.e. we will see the subconscious gestures that coincide with speech, [14]); and (b) participants will respond to the other avatar as if it were a person in the ‘real world’. Our expectations for (a) come from studies suggesting that participants have a sense of ownership over their avatars. For example, the rubber hand illusion demonstrated in VEs, where people feel the virtual arm is their own. This has been demonstrated with tactile stimulation, e.g. [20], and with synchronous movement of a real and virtual hand [21]. The principle from the rubber hand illusion is extended to the whole body in investigations into third-person out-of-body experiences [22]. Further, other studies have reported the importance of kinematic fidelity of the avatar, suggesting it is more important than a visually faithful avatar appearance [23].

Evidence for (b), participants responding to other avatars as if they were real, comes from studies that show this despite participants knowing that they are virtual characters controlled by a computer. This is shown by a virtual Milgram experiment, where participants had to administer electric shocks to a virtual character, and changes in physiological responses were greater compared with a condition where the avatar was not displayed [24]. In another example, participants responded with more anxiety to a negative virtual audience (computer controlled characters) compared with the same characters exhibiting positive nonverbal feedback [25]. Participants' responses to virtual characters have been found to be similar to videos of real people (in the context of participants' change of opinion in a persuasion exercise), and this applies when they were not visually faithful representations, and also when they were non-human virtual characters [26].

It is highly likely that there is a relationship between (a) and (b), that is participants' responses to the ‘other’ avatar will affect how they manipulate their own avatar. This is shown by work on mimicry and interactional synchrony. People mimic the body gestures of others during communication and the flow of movement is rhythmically coordinated with the speech and movement of others [27]–[29]. In light of this relationship we consider our interaction in VR on three levels, *no* nonverbal communication, *unidirectional* and *bidirectional* nonverbal communication (see materials and methods).

In our experiments participants wore a HMD and were given a self-avatar, and they saw the avatar of the other person. The objective of experiment 1 was to determine the importance of the level of nonverbal communication and camera perspective for communicating in VR using a word description task. The results informed us that bidirectional nonverbal communication and third-person camera perspective led to significantly more hand gesturing (compared to other VR conditions) and significantly better performance in the task. In addition, participants gave up describing more words when they were talking to a static avatar (i.e. nonverbal feedback was not available).

The aim of experiment 2 was to further investigate the importance of the nonverbal feedback from the guesser's avatar. It was found that plausible but unintelligent nonverbal feedback from a prerecorded animation was detrimental to task performance, suggesting that participants were influenced by the nonverbal feedback they were receiving. The studies both showed evidence of a gesture suppression effect in HMD VR compared to the same task performed without a HMD. We recommend future work on understanding and alleviating this effect to help with comparisons between HMD VR and real world communication.

## Results and Discussion

### Experiment 1

In the first experiment we manipulated the availability of nonverbal communication for the describer and the guesser, and camera perspective, in a word description task.

The dependent measures obtained were the number of words successfully described, the number of words passed and the amount of movement. Refer to materials and methods for more information.

#### Movement

There was a strong correlation between the speed of movement of left and right hands, Spearman's 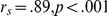, therefore only the dominant hand was used in further analyses. In addition, our hand movement analyses focus on the describer (they were the speaker in this task), and an overall comparison of the describers' and guessers' dominant hand movement confirmed the describer used significantly more hand movement than the guesser, Mann-Whitney's 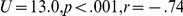.

**Figure 1:**
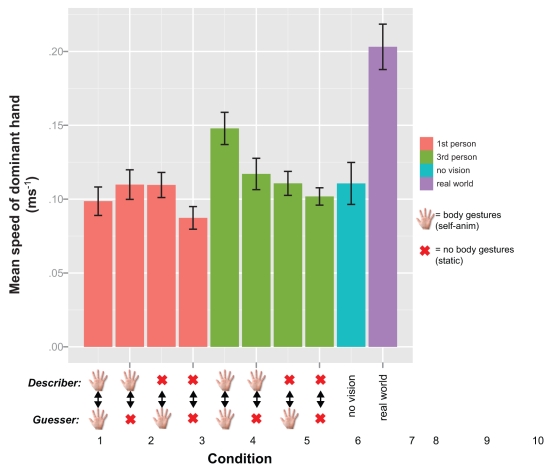
Movement analysis for experiment 1. Mean describer hand movement in each condition. The hand icon is representative of the availability of body gestures for the describer/guesser (see legend). For example, condition 1 is bidirectional nonverbal communication, conditions 2+3 are unidirectional, and in 4 no nonverbal communication is available to either the describer or the guesser. Similar for conditions 5 through 8 (third-person perspective). Conditions 9 and 10 represent no vision (black screen) and real world (without HMD). Error bars represent 1 standard error of the mean.

Figure 1 shows the mean movement of the describer's dominant hand in each condition. Participants gestured almost twice as much in the real world condition compared with the VR conditions (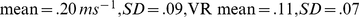). In the VR, participants moved most when both avatars were self-animated and the camera was in third-person perspective (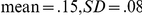). Participants moved least when both avatars were static and the camera was in first-person (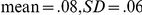).

VR conditions were analyzed with an ANOVA and post hoc tests. The real world conditions and no vision conditions were compared to the VR mean–the former to look for differences between real and virtual world communication, and the latter to observe how the task would be performed without any visual information (see discussion). Participants hand movement was significantly greater in the real world condition compared to the mean of the VR conditions, 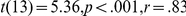. There was no significant difference between the VR conditions and ‘no vision’ 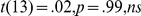.

**Figure 2:**
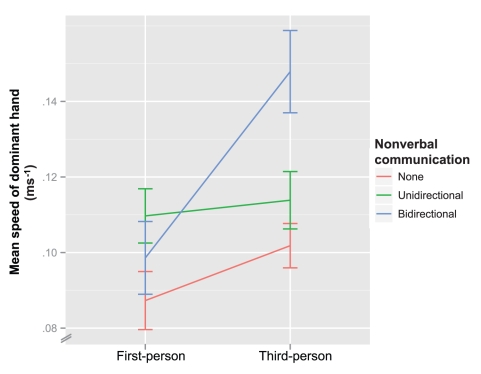
Experiment 1 interactions. Interaction effect between nonverbal communication and camera perspective, for describers' hand movement. Error bars represent 1 standard error of the mean.

A repeated measures ANOVA (DV: mean speed of the describer's dominant hand, IVs: level of nonverbal communication, and camera perspective) showed a significant main effect of camera perspective on the amount of movement, 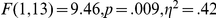. There was a significant main effect of the level of nonverbal communication, 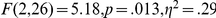. There was a significant interaction effect between camera perspective and level of nonverbal communication, 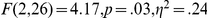. This interaction is shown in Figure 2. A post hoc analysis using paired samples t-tests with Bonferroni correction showed no significant difference between first- and third-person perspective when nonverbal communication was not available 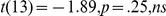, and no significant difference when it was unidirectional 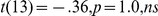, but a significant difference between camera perspectives was found in the conditions with bidirectional nonverbal communication 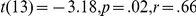. This suggests that camera perspective influences movement more in the bidirectional conditions: movement was greater in bidirectional conditions when the camera was in third-person.

#### Task performance

**Figure 3:**
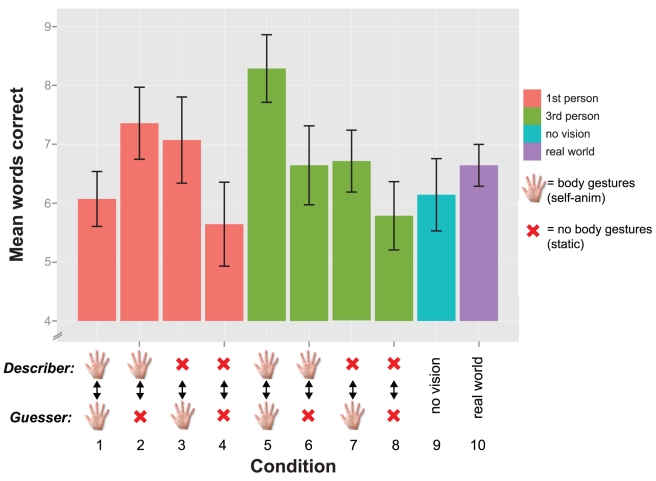
Task performance analysis for experiment 1. Mean number of words successfully described in each experimental condition. Conditions are labeled similarly to Figure 1. Conditions 1 through 4 are first-person perspective. Conditions 5 though 8 are third-person. Error bars represent 1 standard error of the mean.

Descriptively, participants' highest mean performance was observed when both avatars were self-animated, and the camera was in third-person perspective (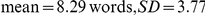). Participants' poorest mean performances occurred when both avatars were static (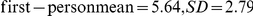; 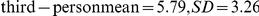). The performance is shown in Figure 3.

A repeated measures ANOVA (DV: mean words successfully described, IVs: level of nonverbal communication, and camera perspective) showed a significant main effect of the level of nonverbal communication on task performance, 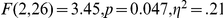. A planned contrast showed a significant difference between no nonverbal communication available (both avatars static) and bidirectional nonverbal communication (both avatars self-animated), 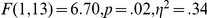.

There was no significant difference between the real world condition and the VR mean, 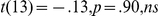, and no significant difference between the no vision condition and the VR mean, 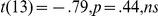.

**Figure 4:**
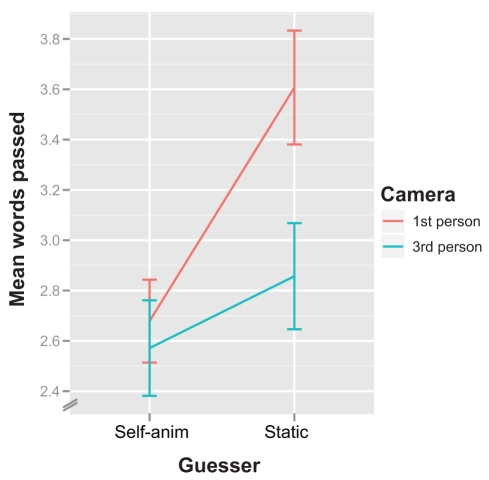
Words passed in experiment 1. The mean number of words passed when the guesser was static and self-animated, in first- and third-person perspectives. Error bars represent 1 standard error of the mean.

A repeated measures ANOVA (DV: mean number of words passed, IVs: guesser's avatar self-animated vs. static, and camera perspective) showed a significant main effect of the guesser's avatar (self-animated vs. static), 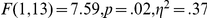. There was also a significant main effect of camera perspective (first- vs. third-person), 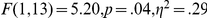. The interaction effect was not significant, 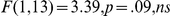. Put simply, participants passed significantly more words in first-person perspective, and significantly more when the guessing avatar was static (Figure 4).

#### Discussion

With the ability to animate virtual characters in real-time in virtual reality, one should first ask whether a person can take advantage of animation for successful communication. We found that people do perform better when both persons in a communication task have an animated self-avatar as compared to a static avatar. However, this advantage did not occur in first-person perspective. We expect this is because of the problem of *awareness*. This is an issue identified in previous work, where participants were not aware of the functionality of the system or their activities within the environment [3], [30]. In our experiment, the low field-of-view of the HMD meant that participants were not aware of their own avatar movements. They would have had to hold their hands up directly in front of the HMD, or look right down at their body to see that they had an avatar and to determine if it was self-animated. If participants did not do this, they would not have been aware of their own avatar, and we would expect the first-person perspective condition with both avatars self-animated to be similar to the first-person condition with only the guesser self-animated (i.e. conditions 1 and 3 should be statistically similar). A paired samples t-test shows that these conditions are not significantly different, 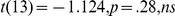, therefore the results do not contradict this hypothesis.

We do not intend to claim from these results that first-person perspective is necessarily ‘worse’ than third-person. Other studies have dealt with the problem of awareness in first-person by using a virtual mirror, so participants could be aware of their own avatar [11], [31]. In our experiment, the third-person perspective compensated for this because participants became aware of their own avatar's movements as soon as they performed any body movement (their avatar was rendered to the HMD at all times), and this is where we see the strongest influence of gestures on performance.

The mean task performance in VR was not significantly different from the no vision or real world conditions, although the movement in real world was significantly greater. This shows that the task can be performed in VR, and taking the mean VR score showed no evidence that participants were worse or better than in the real world. The real world condition also required participants to wear motion tracking markers to monitor movement, and the influence of these is still unknown (they would have been visible to both describer and guesser in the real world condition, but not in VR). It is possible that people would perform better in the game (compared to VR) in a more natural setting, e.g. standing in the real office environment that the avatars inhabited (the rendering was of a real location), without motion tracking equipment or using a less invasive alternative.

In addition, it was important for our hypothesis that we chose a task that participants could perform entirely in the absence of nonverbal communication. The results show that (a) the task could be performed without any visual information and (b) that the presence and absence of body gestures when a body was displayed on the HMD had an effect. We suggest that participants are using a different strategy when no visual information is provided. This hypothesis could be tested by recording the audio communication and making a comparison to the words used in no vision and vision conditions, and investigating this is left for future work.

In summary, the results show that, in VR, people move more and perform better at the task with bidirectional nonverbal communication in third-person perspective. This effect did not occur in first-person perspective with a low field-of-view HMD. The hand movement is at its lowest when both avatars are static, which is in line with work showing that some gestures are produced to benefit the listener [19], and the lower performance in these conditions could also be indicative of a reduced speaker-benefit (we considered this in the introduction). These results are further supported by an increase in the number of words passed (this had a negative impact in terms of task performance: participants lost time) when the camera was in first-person, and in addition when the guessing avatar was static.

The results from this experiment lead to the question of the importance of the listening avatar in third-person perspective. Why does nonverbal communication have to be bidirectional? The describing participants passed significantly more words when the guessing avatar was static. This could be because describers were getting no information regarding the (mis-)understanding of the guessing participant, and gave up more easily. However, was the guessing avatar really giving important nonverbal feedback? Or was it simply important to have them move, and talking to a static avatar was distracting? The importance of nonverbal feedback was further investigated in experiment 2.

### Experiment 2

In the second experiment we investigated the importance of nonverbal feedback from the person listening to us. In one condition we gave listening participants self-animated avatars (as before), and in another we instead animated their avatar with a prerecorded animation. When we talk to someone, is the person listening to us really giving important nonverbal feedback? Or is it simply important to know that they are actively listening?

New participants played the same communication game as in experiment 1. In this experiment we wanted to investigate the importance of nonverbal feedback from the person listening to us, and therefore we varied only the guessers' avatars. The conditions were static, self-animated, prerecorded animation, 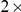 mapping (for hand movement), no vision and real world, and these are described in full in ‘materials and methods’, along with our hypotheses.

The metrics were the same as experiment 1, and were the number of words successfully described and passed, and the hand movement.

#### Movement

As with the previous experiment, there was a strong correlation between the movement of the describers' left and right hands, Pearson's 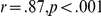, therefore we used the movements of each describer's dominant hand in the analyses. The means and standard deviations for each condition are reported in Table 1.

**Table 1:** Movement analysis for experiment 2.

Describer	Guesser	Mean ( 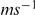 )	SD
Body gestures (self-anim)	No body gestures (static)	.166	.096
Body gestures (self-anim)	Body gestures (self-anim)	.170	.096
Body gestures (self-anim)	Body gestures (prerecorded)	.176	.105
Body gestures (self-anim)	Body gestures ( 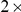 )	.191	.121
No vision		.179	.119
Real world		.260	.154

The mean and standard deviation of the speed of the describer's dominant hand for each condition in experiment 2. The VR manipulations were of the guesser's avatar only.

A comparison between overall describer and guesser dominant hand movement showed that the describer gestured significantly more than the guesser, 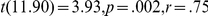.

A repeated measures ANOVA (DV: mean speed of the describer's dominant hand, IV: experimental condition, i.e. static, self-animated, prerecorded animation, 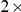 mapping, no vision, real world) showed a significant main effect of condition on describer's hand movement, 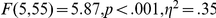. Post hoc tests (using Bonferroni correction for multiple comparisons) showed participants moved significantly more in the real world (without HMD) than in the no vision (black screen) condition, 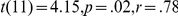. Participants moved significantly more in the real world compared to the 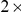 condition, 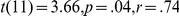. In addition, participants moved significantly more in the real world compared with the VR condition with self-animated avatars, 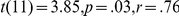. All other pairwise comparisons (with Bonferroni correction) were not significant.

In summary, we replicated our previous findings that describers' hand movements were significantly greater than the guessers', and describers gestured significantly more in the real world condition.

#### Task performance

**Figure 5:**
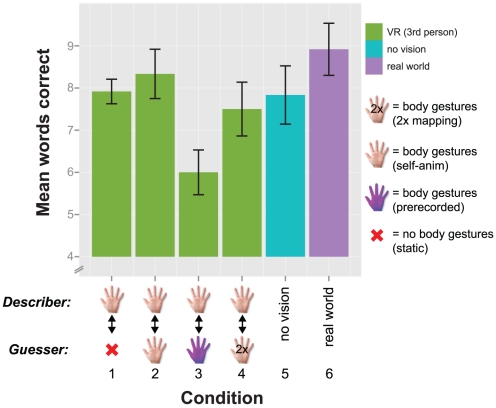
Task performance analysis for experiment 2. Mean number of words correctly described. The VR conditions manipulated the guesser only (the describer could gesture throughout). The hand icons represent body gestures with 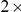 mapping, normal mapping, and body gestures from a prerecorded animation. Error bars represent 1 standard error of the mean.

Figure 5 shows the task performance (mean number of words correctly described) for the conditions in experiment 2. Note that we don't have a condition with both avatars static in this experiment, which was the worst performance in experiment 1. One score collected had a 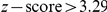 (i.e. greater than 3 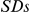 from the mean, therefore an outlier) and was transformed to 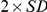.

Descriptively, participants' highest mean performance was observed when both avatars were self-animated, (condition 2, 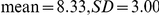). Participants' poorest performance was when the listening avatar was moving based on a prerecorded animation, (condition 3, 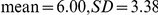).

A repeated measures ANOVA (DV: mean words successfully described, IV: experimental condition) showed a significant main effect of the experimental manipulation 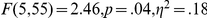. A planned contrast showed a significant difference between a self-animated avatar and an avatar with prerecorded animation (conditions 2 and 3), 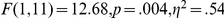. A planned contrast between self-animated avatars with normal 1-1 mapping and the 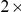 mapping of the guesser's hand gestures was not significant (conditions 2 and 4), 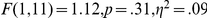. There was no significant effect of experimental manipulation on the number of words passed, 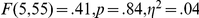.

#### Discussion

Our second experiment supports our hypothesis that listeners' real nonverbal feedback is important for communication and cannot simply be substituted by a non-intelligent animation. Participants performed significantly worse when the guessing avatar was animated with a prerecorded animation instead of his/her own movements. This prerecorded animation was taken from the best pair from experiment 1, so the movements were specific to the game, but did not provide any feedback about understanding or what the speaker should talk about next. Since the no vision condition does not show this significant decrease in performance, it is likely that this prerecorded animation was distracting to the speaker.

In both experiments, hand movement in the no vision condition was not significantly different from the VR conditions. This suggests that people are still gesturing when their movements are not transmitted to the other person (no vision). Evidence that the movements are hand gestures (as opposed to movements that would have occurred in absence of speech) comes from the comparisons between describer and guesser hand movement. In both experiments these comparisons confirmed the describer (who inevitably does most of the talking in the word description task) used significantly more hand movements than the guesser, see Table 2 for a summary. Further, the non-significant difference between VR movement and movement in the no vision condition is consistent with work showing that the usage of gestures from blind talking to the blind is similar to sighted-to-sighted [32].

**Table 2:** Overall describer and guesser hand movement.

	Experiment 1		Experiment 2	
	Mean ( 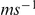 )	SD	Mean	SD
Describer	.120	.084	.179	.109
Guesser	.045	.018	.066	.036

Mean and standard deviation of the describers' and guessers' dominant hand movement across both experiments.

The increased hand movements in the real world did not correspond to a greater task performance, and we are consistent with our discussion of experiment 1 when we suggest that the ‘real world’ condition was arguably not a very natural setting. Participants may have been distracted by the motion tracking markers of themselves and their partner, which were otherwise hidden by the HMD (see materials and methods). An alternative motion tracking setup for future work is considered in our conclusions.

In the case of our guesser, the 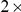 exaggerated condition did not show a significant effect. Note that this only exaggerated hand movements, and a fairer test would probably include the exaggeration of all body movements (e.g. head orientation), which is non-trivial due to movement artifacts (i.e. movements appearing unnatural). Having said this, resulting avatar hand movement was fairly similar in the self-animated 1-1 mapping and 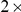 mapping conditions (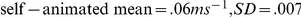; 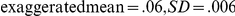). This could be indicating that participants used their avatar like a puppet, reducing their own movements to achieve the desired effect in their third-person avatar. If this is true, one cannot simply exaggerate VR movements to bring them to real world levels.

Interestingly in experiment 2, participants' overall performance was better than in experiment 1. The reader should note that experiment 2 was conducted using native German speakers while the first experiment was conducted using native English speakers, and therefore German verbs and English verbs respectively (see ‘materials and methods’). This limits the usefulness of direct comparisons between the two sets of results, but with this in mind we suspect that the German verbs were easier than the English verbs for describing and conveying the meaning to the other participants.

In experiment 2 we conducted the condition with bidirectional nonverbal communication (both avatars self-animated). We see the same trend (this was participants’ highest scoring VR condition), but not a significant difference. It is important to note that in this experiment we did not repeat the condition without the availability of nonverbal communication (both avatars static), where the largest difference was seen. In summary, our main result is that when the listening avatar had a prerecorded animation, performance in the communication task suffered.

### Conclusions

Virtual reality is a very promising media for further understanding communication, and specifically for evaluating the relative importance of different information channels on successful communication. In this paper we have described two experiments which have asked first whether HMD VR is a good technology to investigate the influence of body gestures in communication and second whether body gestures are used to help in communicating the meaning of a word. More specifically we were interested in understanding the importance of gestures from both the describer and the guesser. In both experiments, describers' hand movement (a simple measure of gestures) was much larger in the real world conditions compared with VR: almost twice as much for experiment 1, and 1.5 times larger in experiment 2. Note that in the ‘real world’ conditions, participants still wore the motion tracked objects (on their hands, shoes, head and backpack). We do not know how these objects affected participants' movements (we would need to use an alternative body tracking technology to determine this), but we do know that wearing a HMD made a difference. Evidence of the importance of gestures from our studies and from the literature highlights the need to understand the cause of this gesture suppression effect in HMD VR. Future work on alleviating this effect should help us create a VR scenario in which participants behave in a more similar way to the real world, and in which one could therefore learn more about interpersonal communication.

We found that people move more and perform better in a communication task in HMD VR when they have a third-person perspective view of a self-animated avatar for both the describer and guesser. We further found that movement of the guessing avatar was not sufficient for this increase in performance, but rather that the animation needs to be coupled to the real movements of the listening participant. This finding is particularly important to the development of virtual humans: automated characters that simulate listening behavior, e.g. [13]. Further work would be required to determine what types of gestures are most important, and to tease apart the contribution of posture change, hand movements and facial expressions to communication in VEs.

Our dependent measure in the main analysis was the resulting score from each pair in each condition, which represents a two-way communication which is affected by the presence and absence of gestures in VR. The finding that bidirectional nonverbal communication is important is consistent with work reporting interaction synchrony as explained in the introduction. Whether gestures helped the guesser infer or the describer explain would require a new paradigm, where participants think their gestures can be seen but they are not transmitted, i.e. using the concept of transformed social interaction [33]. Hence, our conclusions are not specifically applied to one of either the describer or the guesser, but to the communicating pair. We show that enabling self-animated avatars for the pair improves communication in head-mounted display virtual environments, we suggest that they need to be aware of the functionality of their avatar and the availability of body movements, and they need feedback from the other that cannot be substituted by unintelligent animation.

Our suggestions for future work are to investigate communication in VEs without a head-mounted display. The HMD was causing gesture suppression, therefore one would expect a stronger effect without it, e.g. using immersive projection technology as an alternative. In addition, one can conduct further research where participants wear less obtrusive objects on their hands and feet, e.g. using inertial motion tracking from Xsens [34], or the Microsoft Kinect camera and depth sensor [35]. Since our system runs over a network, it would also be possible to investigate cross-cultural differences in nonverbal communication by modifying it to run over the Internet, where participants are located in different countries. We predict an increase in research with self-animated avatars for both communication and interaction purposes. This paper lays the foundation for how one might investigate communication in an immersive VE.

## Materials and Methods

### Experimental setup

**Figure 6:**
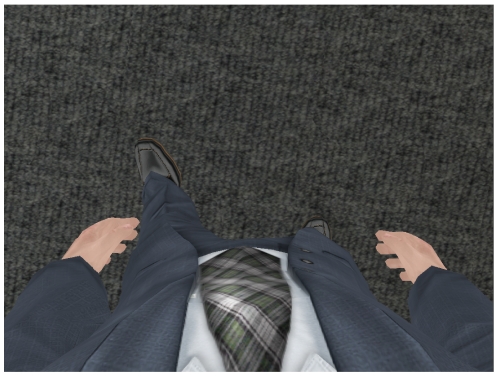
First-person self-avatar. Example of first-person perspective, looking down at the avatar's body.

**Figure 7:**
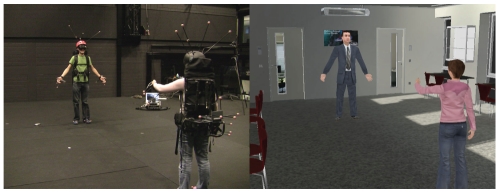
The experimental setup. Left: The participants wore a total of six tracked objects (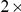 hands, 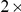 feet, backpack and helmet). Right: the corresponding virtual environment, showing the avatars in the self-animated third-person perspective.

Participants' body movements were tracked using an optical tracking system (16 Vicon MX13 cameras) and mapped onto a self-avatar in real time. For example, in first-person perspective, if participants held up their real hands in front of their eyes, they saw their avatar's hands in the HMD, and if they looked down then they saw their avatar's body (see Figure 6). The third-person camera looked over the shoulder of the participant's own avatar, and was positioned so that they could also see the other avatar (Figure 7).

Participants each wore a total of six rigid-body objects that were tracked, placed on their hands, feet, backpack and helmet (Figure 7 shows the objects and corresponding third-person perspective). The objects on their hands were attached across the palm and the wrist. Participants could put the palms of their hands together, but the markers restricted certain gestures close to the body (e.g. participants could not fold their arms). Note also that facial expression and eye gaze were not captured, due to the use of a HMD.

The virtual reality setup was implemented in Virtools 4.1 from Dassault Systèmes. The positions of all joints which were not tracked were calculated using built-in inverse kinematic algorithms, and in addition a calibration was applied which scaled the avatar to the height of the participant.

**Figure 8:**
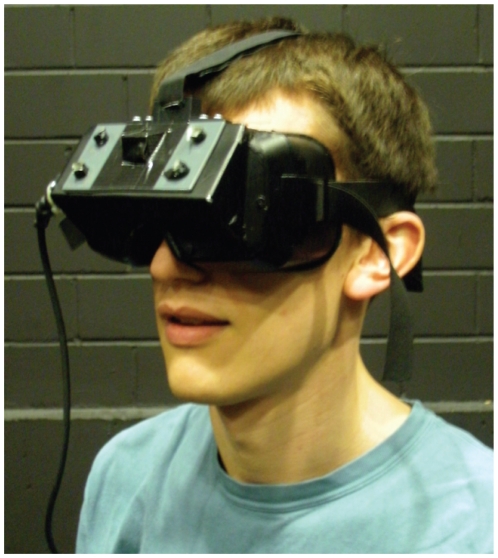
Head-mounted display. Participants wore an eMagin HMD mounted in goggles, so they could see the virtual world and not the real world.

The participants were given a male or female avatar to match their gender. The environment was an office room (10 m length, 6.80 m width, 2.77 m height) and was symmetrical (left/right walls and front/back walls were the same), apart from the main light source which came from one side only (Figure 7). Participants stood 4 m apart, and each viewed the scene using a light-weight head-mounted display (eMagin Z800 3D Visor, mono, Figure 8) that provided a field of view of 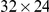 degrees at a resolution of 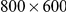 pixels for each eye.

Due to the number of objects tracked in close proximity (six objects per person), we increased the delay of the tracking software for higher tracking accuracy. The end-to-end delay of the whole experimental setup was measured as approximately 150 ms using light sensing techniques developed in [36].

### Communication task

Inspiration for our communication task came from foreign language learning, where students infer the meanings of new words from teachers' descriptions which take place in the foreign language itself (as opposed to providing a direct translation). This is a language-learning strategy known as inductive learning [37]. The full rules for the task are described in the method below.

### Experiment 1

#### Participants

A total of 14 participants (8 male and 6 female) with a mean age of 26.6 (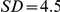) took part in the study. We advertised for pairs of participants and all participants applied to take part with someone they knew. All participants spoke English as their first language. All participants volunteered for the experiment, gave informed written consent (they signed a consent form that gave them information about the task and motion tracking setup without disclosing hypotheses), and were paid standard rates for their participation. These experiments were performed in accordance with the 1964 Declaration of Helsinki and were approved by the ethical committee of the university hospital of Tübingen. Participants were debriefed and informed of the purpose of the study at the end of the experiment.

#### Hypothesis

Our hypothesis was that participants would perform better in the communication game in HMD VR when they had nonverbal communication available (body gestures, avatars self-animated), compared with no nonverbal communication available (no body gestures, avatars static).

#### Independent variables

We manipulated camera perspective (first- vs. third-person) and level of nonverbal communication. We use terminology from media communication literature to describe three levels of nonverbal communication as: (1) no nonverbal communication available, (2) *unidirectional* and (3) *bidirectional* nonverbal communication [38]. The terms unidirectional and bidirectional are usually used in the literature to describe a given communication medium as a whole. For example, television is a unidirectional medium (you cannot talk back to the presenter), and a video conference is bidirectional. In our case, we were not manipulating the entire medium, we were changing one aspect of it (nonverbal communication) and verbal communication remained bidirectional throughout. Nonverbal communication was applied to avatars using motion tracking (described above) and in conditions where it was not available participants' avatars were frozen in a neutral pose.

In addition, data were collected with a ‘no vision’ (black screen) condition and a real world condition (where participants played without a head-mounted display, but still wore the markers to collect tracking data).

#### Method

Participants were given written and verbal instructions on how to play the communication game, including an example. A training phase involved first playing the game in the real world, face-to-face, before putting on the virtual environment equipment. Then participants played two practice rounds in VR, taking turns to be the describer.

The game was played in rounds of three minutes, with one person as the describer and one person as the guesser in each round. The describer was given words on the screen by the experimenter, and the guesser had to shout out the correct answer. Each time the word was guessed (or passed), the experimenter provided a new word to the HMD (via button press), and the status of the word (guessed or passed) was automatically saved to a log file. Experimenter's judgments were not blind to condition. Participants were instructed to try to successfully describe as many words as possible in three minutes. At the end of the round their score was displayed on the screen. Participants were given a break half way through the experiment.

The study was a repeated-measures design, and the condition was changed each round. The 10 conditions were presented once for each participant, and were counterbalanced by randomizing the order across pairs.

Describers were not allowed to say what letters were in the word, or how many letters it had. They were not allowed to say the word itself, or any derivative (e.g. if the word was ‘swim’, they could not say ‘swimmer’ or ‘swimming’). They were not allowed to use ‘rhymes with’ or ‘sounds like’ clues.

Describers were allowed to use gestures, act and mime the word. They were allowed to pass words.

The words to be described in the game were randomly selected from the top 1000 verbs in the British National Corpus [39]. The words were tagged using TreeTagger software [40].

#### Movement analysis

**Figure 9:**
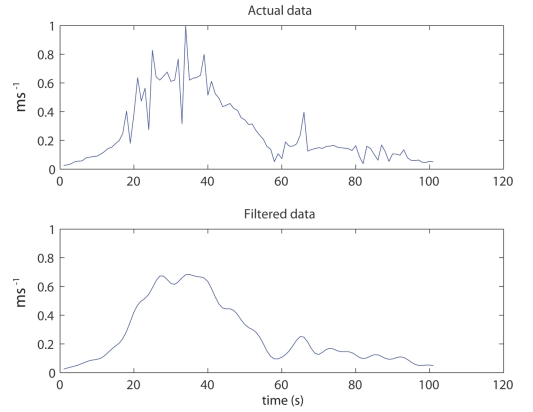
Movement analysis method. An example of before and after filtering motion data. The first 100 seconds (x axis) from one participant is shown. The y axis represents the speed of movement of their dominant hand.

To get a quantitative measure of the amount of nonverbal gesturing the position of participants' hands were recorded at 60 Hz. A Butterworth filter was applied to the real motion tracking data (Figure 9). These data were used to calculate the average speed of participants' hands.

The Shapiro-Wilk test was significant for right hand movement, 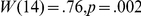, and for left hand movement, 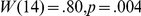, and so Spearman's 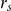 was reported for the correlation. The Shapiro-Wilk test was significant for describer movement, 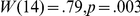, and for guesser movement, 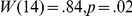, and Levene's test indicated unequal variances, 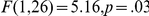, therefore the comparison between describer and guesser movement was performed using a Mann-Whitney 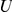 test.

### Experiment 2

#### Participants

A total of 12 participants (5 male and 7 female) with a mean age of 25.8 (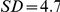) took part in the study. Participants had not taken part in experiment 1. Participants were each paired with someone they knew. All participants spoke German as their first language, volunteered for the experiment, gave informed written consent (they signed a consent form as in experiment 1), and were paid standard rates for their participation. They were debriefed at the end of the experiment.

#### Hypotheses

Our hypothesis was that we receive useful nonverbal feedback from the person we speak to, and therefore the primary conditions for comparison in this experiment were (1) talking to a self-animated avatar, and (2) talking to an avatar with a plausible *prerecorded* animation to simulate listening behavior. If participants were receiving useful nonverbal feedback, our hypothesis was that a prerecorded animation would be detrimental to task performance. In other words, the guessing avatar would act realistically, but the nonverbal communication would be false – the guessing avatar would nod, but at the wrong time; it would look away to indicate thinking, but when the participant was actually doing something else.

Our hypothesis is in line with previous research in VEs that demonstrates people's sensitivity to desynchronized body gestures in prerecorded conversations. McDonnell and colleagues had audio and body motions of small group conversations recorded in a motion capture session, and played them back to participants using avatar representations. Conversations with gestures misaligned in time or played back from different conversations entirely were noticed by participants, who determined them to be less realistic compared with conversations with body gestures from the original recording [41], [42]. In our study, the investigation is from the point of view of a participant in the communication, as opposed to an observer of a prerecorded crowd scene.

Finally, an exploratory condition investigated ‘exaggerated’ hand gestures, where a 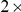 mapping was applied. Our hypothesis was that if hand gestures from the guesser are important, exaggerating them would make them more noticeable, and potentially increase task performance.

#### Independent variables

The conditions varied the guesser's avatar, and were static, self-animated, prerecorded animation, and 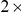 mapping (for hand movement), no vision and real world.

To make our guessing avatar's prerecorded condition plausible (and therefore a difficult test for our hypothesis), we took the animation from the best guesser of experiment 1. This means that in the prerecorded condition the guesser's avatar moved exactly how the guesser moved for the highest scoring pair in experiment 1 – the animation was plausible listening behavior, but did not correspond to the real movements of the guessing participant.

**Figure 10:**
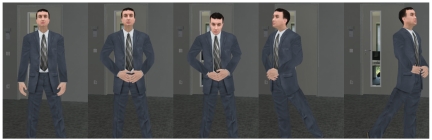
Prerecorded animation in experiment 2. Extract of prerecorded animation. Left to right: (1) Neutral pose; (2) with hands together; (3) looking down; (4) turning right; (5) turning left.

The animation included gaze towards the other participant in a neutral pose, changing gaze direction, putting hands together, changing posture and stepping side-to-side. An example of part of the animation is shown in Figure 10.

The 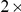 mapping worked as follows: if the participant moved their hand 5 cm, the avatar moved its hand 10 cm in the same direction. The 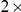 condition was calibrated so participants could put their hands together and their avatar's hands and arms would not intersect. To achieve this, the mapping started from the center of the participant (pelvis position).

Due to the problems of awareness of one's own avatar in first-person perspective, all VR conditions in experiment 2 were conducted in third-person.

#### Method

The method was the same as experiment one, except for the words used. Experiment 2 was conducted in German, and the words were taken from the DeWaC corpus [43].

#### Movement analysis

A Butterworth filter was applied to the real motion tracking data as before. Levene's test indicated unequal variances between the describer and guesser movement, 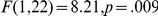, therefore the Welch approximation to the degrees of freedom was used for the comparison.
